# Revealing the Role of Alternariol in the Local Steroidogenesis in Human Prostate Normal and Cancer Cells

**DOI:** 10.3390/ijms24119513

**Published:** 2023-05-30

**Authors:** Kinga Anna Urbanek, Karolina Kowalska, Dominika Ewa Habrowska-Górczyńska, Marta Justyna Kozieł, Kamila Domińska, Agnieszka Wanda Piastowska-Ciesielska

**Affiliations:** 1 Medical University of Lodz, Department of Cell Cultures and Genomic Analysis, 90-752 Lodz, Poland; kinga.urbanek@umed.lodz.pl (K.A.U.); karolina.kowalska1@umed.lodz.pl (K.K.); dominika.habrowska@umed.lodz.pl (D.E.H.-G.); marta.koziel@umed.lodz.pl (M.J.K.); 2 Medical University of Lodz, BRaIn Laboratories, 92-216 Lodz, Poland; 3 Medical University of Lodz, Department of Comparative Endocrinology, Zeligowskiego 7/9, 90-752 Lodz, Poland; kamila.dominska@umed.lodz.pl

**Keywords:** alternariol, steroidogenesis, mycotoxin, carcinogenesis, dehydroepiandrosterone

## Abstract

The mycotoxin alternariol (AOH) can be found in food products infected by *Alternaria* spp. and is considered an endocrine-disruptive mycotoxin. The main mechanism of AOH toxicity is associated with DNA damage and modulation of the inflammation process. Still, AOH is considered as one of the emerging mycotoxins. In this study, we have evaluated how AOH might affect the local steroidogenesis process in the prostate, in both normal and cancer cells. We have found that AOH itself modulates the cell cycle, inflammation, and apoptosis, rather than the steroidogenesis process in prostate cancer cells; however, in the presence of another steroidogenic agent, the influence on steroidogenesis is significant. Therefore, this is the first study to report the effect of AOH on local steroidogenesis in normal and prostate cancer cells. We postulate that AOH might modulate the release of the steroid hormones and expression of the key components by interfering with the steroidogenic pathway and might be considered a steroidogenesis-altering agent.

## 1. Introduction

Mycotoxins are secondary metabolites of *Aspergillus*, *Fusarium*, and *Penicillium* fungi [1]. The Food and Agriculture Organization (FAO) estimates that up to 25% of global food crops are contaminated by mycotoxins [2]. These chemicals pollute food commodities worldwide, posing a number of significant food safety concerns.

Alternariol (AOH) is one of mycotoxins produced by *Alternaria* species and is often found in vegetables as well as fruits and processed fruit products (i.e., juices, wine) [3]. The presence of *Alternaria* mycotoxins has been widely reported; however, they still serve as emerging mycotoxins, for which a detailed molecular mechanism for toxicity has not been determined [4]. The European Food Safety Authority (EFSA) assessed that 2.4–31% of grain samples are contaminated with AOH. Dietary exposure to AOH was estimated in toddlers at 3.8–71.6 ng/kg bodyweight per day [5]. This fact has raised public concern about the relevance of the cytotoxic and genotoxic potential of *Alternaria* mycotoxins, as well as the need for a detailed determination of their health consequences [6].

AOH has been considered as an endocrine-disruptor chemical (EDC) due to its reported binding to estrogen and androgen receptors in cells. The acute toxicity of AOH is regarded as low; however, little is known about the biological effects of AOH on reproduction, and the detailed cellular molecular mechanisms still need to be established [7]. AOH exposure results in increased expression of progesterone receptor (PR) and modulation of the production of estradiol (E2) and progesterone in the H295R cell line [8]. The estrogenicity of AOH is considered weak and partial; however, there are reports that indicate AOH to be an androgen receptor (AR) agonist. It was also suggested that AOH might modulate the endocrine system through interference of nuclear receptor signaling or upregulation of the expression of steroidogenic receptors [9]. Most importantly, it was postulated that AOH can mimic cholesterol in cells and thus alter the hormonal balance [10]. 

Prostate cancer (PCa) is considered as the second leading cause of male cancer death [11]. The increasing average age at the time of diagnosis, being 66 years, correlates with the morbidity and mortality of prostate cancer worldwide [12]. Androgen deprivation therapy is the keystone treatment for men with advanced metastatic PCa as the prostate gland growth and development is dependent on androgens [13]. Majority of deaths from prostate cancer are the result of castration-resistant prostate cancer (CRPC). The potent androgens were confirmed in subsequent studies in patients after the surgical castration [14]. Compared to the pre-treatment level, the residual concentrations of intra-tumoral dihydrotestosterone (DHT) can reach from 10% to 40%, while reaching the castration concentration of testosterone with a tumor response in 80–90% of patients [13]. 

Steroidogenesis is a multistep process of steroid hormone biosynthesis from cholesterol. The process begins with the transfer of cholesterol from the intracellular storage to the inner mitochondrial membrane, where it is transformed to pregnenolone and into downstream steroids. As the prostate gland tissue is not capable of the de novo steroid synthesis, the intracellular steroid hormone level depends on the blood delivery and local biosynthesis [15]. Research showed that even after androgen deprivation therapy (ADT), tumor growth still depends on androgen supply, and the recurrence of the tumor is caused by local steroidogenesis in the prostate tumor cells [16]. The crucial role of cholesterol in PCa carcinogenesis was also suggested, as it might be confirmed by a number of studies confirming the usefulness of statins in prostate cancer therapies [17]. An increased level of steroidogenic enzymes that synthesize androgens from cholesterol or other circulating steroid precursors, such as progesterone or dehydroepiandrosterone (DHEA), was reported in PCa [18]. DHEA is an active androgen precursor (testosterone and dihydrotestosterone). It was reported that despite that DHEA is weakly active as androgen receptor ligand, it is largely inactive as an androgen. DHEA might be associated with prostate cancer carcinogenesis, although its cancer progression is unclear [19].

In our previous research, exposure to deoxynivalenol mycotoxin (potentially involved in local steroidogenesis modulation) alone or in combination with DHEA had a stimulatory effect on the release of steroid hormones and the expression of genes related to steroidogenesis [20]. Therefore, it is highly possible that EDC, which modulates the process of steroidogenesis, might also modulate the local steroidogenesis in prostate cells and participate in both initiation as well as progression of the tumor.

In this study, we have evaluated the ability of AOH to alter local steroidogenesis in prostate cancer and normal cells. In the presented experiments, we decided to use the androgen-independent human prostate cancer cell line PC3 (bone-derived metastasis) and the non-cancerous epithelial cell line PNT1A, that has been proven to be a good model for the analysis of cellular processes (i.e., epithelium proliferation in response to androgens and growth factors).

The objective of this study was to determine the effect of AOH alone, as well as in combination with the known steroidogenic agent DHEA, on the production of estradiol, testosterone, and progesterone in prostate normal epithelial cells, as well as adenocarcinoma cells with reported insensitivity to androgens.

## 2. Results

### 2.1. AOH Modulates the Viability of Prostate Normal and Cancer Cell Lines in a Dose- and Time-Dependent Manner

**Figure 1:**
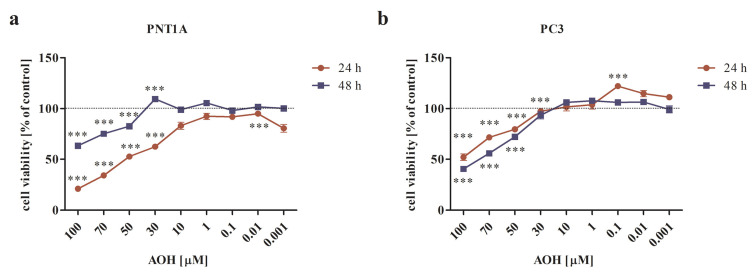
AOH decreases the viability of prostate cells in a dose- and time-dependent manner in normal (**a**) as well as prostate cancer (**b**) cells, as shown with the MTT assay. Results are expressed as mean ± SE of % of control cell viability (control = 100%). *** *p* < 0.001, as compared to the control. AOH—alternariol.

First, the cytotoxic effect of AOH was verified in a dose- and time-dependent manner. The tested dose range was similar to our previous study [21]; however, a different cell culture medium influenced the response of normal prostate cells to AOH. Therefore, the experiments were conducted, and data are hereby shown again. It was observed that AOH significantly affected the viability of both normal and cancer cells in doses higher than 30 µM upon 24 and 48 h of exposure (Figure 1a,b) (*p* < 0.001). Interestingly, we observed that for normal prostate cells, longer incubation times decreased the cytotoxic effect of AOH (Figure 1a), which was not observed for PC3 cells. Based on these results and our previously published data [20], for the rest of the experiments, two doses of AOH have been chosen: 10 µM and 0.1 µM, and one incubation time: 48 h.

### 2.2. AOH Modulates the Local Steroidogenesis in Prostate Normal and Cancer Cells in the Presence of DHEA

**Figure 2:**
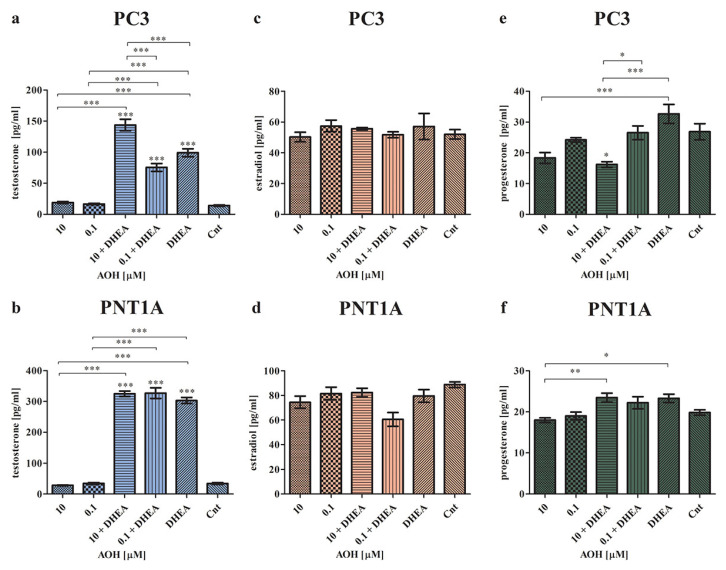
The secretion of testosterone (**a**,**b**), estradiol (**c**,**d**), and progesterone (**e**,**f**) after 48 h of exposure to AOH and DHEA, evaluated by ELISA tests in PC3 and PNT1A cells. Results are presented as mean ± SE. One-way ANOVA was used to perform statistical analysis of the results, and *p* < 0.05 was considered statistically significant. * *p* < 0.05, ** *p* < 0.01, *** *p* < 0.001. AOH—alternariol, DHEA—dehydroepiandrosterone, Cnt—control.

To assess the influence of AOH on local steroidogenesis, cells were treated with both AOH and DHEA, and the production of testosterone, estradiol, and progesterone was assessed (Figure 2). As expected, the highest changes were found for the production of testosterone. Furthermore, we observed that in the case of cancer cells, AOH had a higher impact on the chosen hormones’ production. In both normal as well as cancer cell lines, AOH itself did not change the production of testosterone (Figure 2a,b), although in the presence of DHEA, which itself significantly increased the production of testosterone (*** *p* < 0.001) as compared to control cells, AOH modulated the production of testosterone. In PC3 cells, a statistically significant increase in the production of testosterone was observed for 10 µM of AOH + DHEA, as compared to the control (*** *p* < 0.001), 10 µM of AOH (*** *p* < 0.001), DHEA (*** *p* < 0.001), and 0.1 µM of AOH + DHEA (*** *p* < 0.001). Here, 0.1 µM of AOH + DHEA also significantly increased the production of testosterone as compared to the control (*** *p* < 0.001) and 0.1 µM of AOH (*** *p* < 0.001), but the effect was lower than that caused by DHEA itself. In the normal prostate cell line, both doses of AOH and DHEA caused a significant increase in testosterone production in comparison to the control (*** *p* < 0.001) and treatments with AOH alone (*** *p* < 0.001). No significant changes were observed between the treatments with AOH + DHEA and DHEA, indicating that in the case of PNT1A cells, the observed effect of AOH + DHEA is primarily dependent on DHEA. Next, we evaluated the production of estradiol and progesterone. We did not observe significant changes in estradiol production in both cancer and normal cells (Figure 2c,d). Only a slight decrease in the production of estradiol was observed in PNT1A cells for the 0.1 µM AOH + DHEA treatment, but it was not statistically significant. The last evaluated hormone was progesterone. In the PC3 cell line, we observed that treatment with AOH insignificantly decreased the production of progesterone, as compared to control cells, whereas DHEA itself insignificantly increased it (Figure 2e). The 10 µM AOH + DHEA treatment significantly decreased the production of progesterone as compared to the control (* *p* < 0.05), DHEA alone (*** *p* < 0.001), as well as 0.1 µM of AOH + DHEA (* *p* < 0.05). Similarly, in normal PNT1A cells, AOH also did not significantly modulate progesterone production as compared to control cells (Figure 2f). A statistically significant decrease was observed for 10 µM of AOH compared to the 10 µM AOH + DHEA (** *p* < 0.01) and DHEA treatments (* *p* < 0.05).

**Figure 3:**
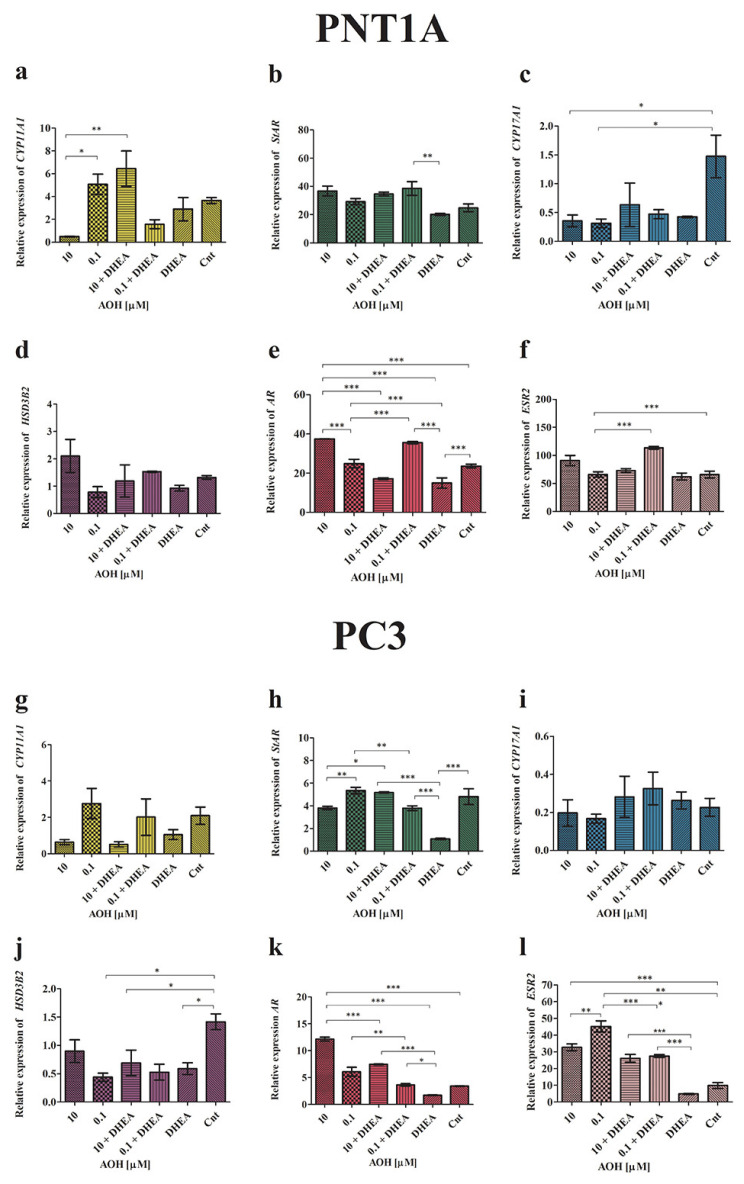
The relative expression of the mediators of the initial and rate-limiting steps in steroidogenesis—*CYP11A1* (**a**,**g**)*, STAR* (**b**,**h**), *CYP17A1* (**c**,**i**)*, HSD3B2* (**d**,**j**), *AR* (**e**,**k**), and *ESR2* (**f**,**l**) obtained by RT-qPCR. The results are expressed as the mean relative expression of three independent experiments (±SE). The results were calculated using the ΔΔCt method. One-way ANOVA was used to perform statistical analysis of the results, and *p* < 0.05 was considered as statistically significant. * *p* < 0.05, ** *p* < 0.01, *** *p* < 0.001. AOH—alternariol, DHEA—dehydroepiandrosterone, Cnt—control, *CYP11A1*—cytochrome P450 family 11 subfamily A member 1, *StAR*—steroidogenic acute regulatory protein, *CYP17A1*—cytochrome P450 family 17 subfamily A member 1, *HSD3B2*—hydroxy-delta-5-steroid dehydrogenase 3 beta- and steroid delta-isomerase 2, *AR*—androgen receptor, *ESR2*—estrogen receptor beta.

Next, we evaluated the expression of the mediators of the initial and rate-limiting step in steroidogenesis: cytochrome P450 family 11 subfamily A member 1 (*CYP11A1*)*,* steroidogenic acute regulatory protein (*StAR*)*,* cytochrome P450 family 17 subfamily A member 1 (*CYP17A1*), hydroxy-delta-5-steroid dehydrogenase, 3 beta- and steroid delta-isomerase 2 (*HSD3B2*), androgen receptor (*AR*), and estrogen receptor beta (*ESR2*) (Figure 3).

Firstly, it was found that AOH modulates the expression of *CYP11A1* in PNT1a cells: 10 µM of AOH decreased its expression, whereas 0.1 µM of AOH increased it, and the effect was significantly different when compared between these groups (* *p* < 0.05). The observed contradictory effect was potentiated by the addition of DHEA: treatment of cells with 10 µM of AOH + DHEA significantly increased the expression of *CYP11A1* as compared to 10 µM of AOH alone (** *p* < 0.01) as well as 0.1 µM of AOH + DHEA (** *p* < 0.01). In case of PC3 cells, a similar, although not statistically significant, effect was observed. The higher tested dose of AOH decreased the expression of *CYP11A1,* and the addition of DHEA boosted that effect. For a lower dose of AOH, no such effect was observed. In PNT1A cells, AOH insignificantly increased the expression of *StAR*, whereas a significant increase was observed for 10 µM of AOH + DHEA as well as 0.1 µM of AOH + DHEA, as compared to DHEA treatment alone (*p* < 0.05 and *p* < 0.01, respectively). The highest increase was observed for 0.1 µM of AOH + DHEA, which was significantly different from the control (* *p* < 0.05). In PC3 cells, DHEA significantly reduced the expression of *StAR* as compared to the control (*** *p* < 0.001). The higher tested dose of AOH slightly decreased, whereas the lower dose increased, the expression of *StAR* as compared to non-treated cells. A different effect was observed for the treatment with 10 µM of AOH + DHEA, which significantly increased the expression of *StAR* as compared to AOH treatment alone, as well as DHEA (*** *p* < 0.001 and * *p* < 0.05, respectively). A contradictory and significant effect was observed for 0.1 µM of AOH + DHEA (*** *p* < 0.001 and ** *p* < 0.01, respectively). In the case of *CYP17A1* expression evaluated in PNT1A cells, the expression in all treatments was decreased as compared to control cells, although significant only for separated treatments of AOH (* *p* < 0.05). A contradictory, yet not significant, effect was observed in PC3 cells, where treatment with DHEA as well as AOH + DHEA resulted in the increased expression of *CYP17A1*. The expression of *HSD3B2* was modulated by AOH. In PNT1A cells, an increased expression for a higher tested dose of AOH was observed as compared to control and DHEA alone, however it was not statistically significant. In PC3 cells, a contradictory effect was observed: all tested doses decreased the expression of *HSD3B2*, whereas statistical significance was observed for 0.1 µM of AOH, DHEA, and 0.1 µM of AOH + DHEA, as compared to the control (* *p* < 0.05). In the last analysis, we evaluated the expression of *AR* and *ESR2* genes. In PNT1A, a statistically significant increase in the expression of *AR* was observed after treatment with 10 µM of AOH, as compared to the control as well as DHEA (*** *p* < 0.001). The addition of DHEA to 10 µM of AOH resulted in a significant decrease in the expression of *AR* as compared to 10 µM of AOH and the control (* *p* < 0.05). A contradictory effect was observed for a lower tested dose of AOH, which itself only slightly increased the expression of *AR,* whereas simultaneous treatment with DHEA resulted in a significant increase in the expression of *AR* as compared to 0.1 µM of AOH (*p* < 0.001), DHEA (*** *p* < 0.001), as well as the control (*** *p* < 0.001). In cancer cells, AOH at a dose of 10 µM caused the highest increase in the expression of *AR* as compared to the control and the DHEA treatment (*** *p* < 0.001). The addition of DHEA to 10 µM of AOH resulted in a decrease in the expression of the tested gene, but still presented a significantly higher expression as compared to the control (*** *p* < 0.001) and DHEA (*** *p* < 0.001). A similar effect was observed for a lower tested dose of AOH, but to a lower extent than that for 10 µM, as compared to 0.1 µM of AOH (** *p* < 0.001) and DHEA (*** *p* < 0.001). The evaluation of the *ESR2* gene also showed that AOH at a dose of 10 µM affected the tested gene in both normal as well as cancer cell lines. In PNT1A cells, we observed a non-significant increase after treatment with 10 µM of AOH compared to the control, whereas treatment with 0.1 µM of AOH + DHEA resulted in a significant increase in the expression of *ESR2* as compared to 0.1 µM of AOH alone (*** *p* < 0.001), DHEA (*** *p* < 0.001), as well as the control (*** *p* < 0.001). In PC3 cells, the effect of AOH was also observed both in separated treatments as well as co-treatments with AOH. Both doses of AOH resulted in an increased expression of *ESR2* as compared to the control (*** *p* < 0.001) and DHEA (*** *p* < 0.001). The treatment with AOH + DHEA also increased the expression of *ESR2*, but to a significantly lower extent than AOH alone. Moreover, a similar effect of DHEA in PC3 cells was observed in the case of the expression of both tested receptors: DHEA significantly decreased the expression of *AR* and *ESR2* as compared to non-treated cells.

### 2.3. AOH Affects the Expression of Caveolin-1 (CAV-1) in Prostate Cells

Caveolins (CAVs) are components of the caveolae, a part of lipid rafts which bind cholesterol and participate both in the process of steroidogenesis and signal transduction. CAV-1 is involved in many cellular processes, such as cell cycle regulation, endocytosis, signal transduction, and cholesterol trafficking and efflux [22]. It has also been suggested that CAV-1 may act as a scaffolding protein responsible for organization and concentration of signaling molecules within caveolae [23]. In prostate cancer progression, an increased level of CAV-1 was reported in tumor epithelial cells [24]. The reverse effect was observed in stromal cells in advanced metastatic prostate cancer, where the expression of CAV-1 was decreased [25].

**Figure 4:**
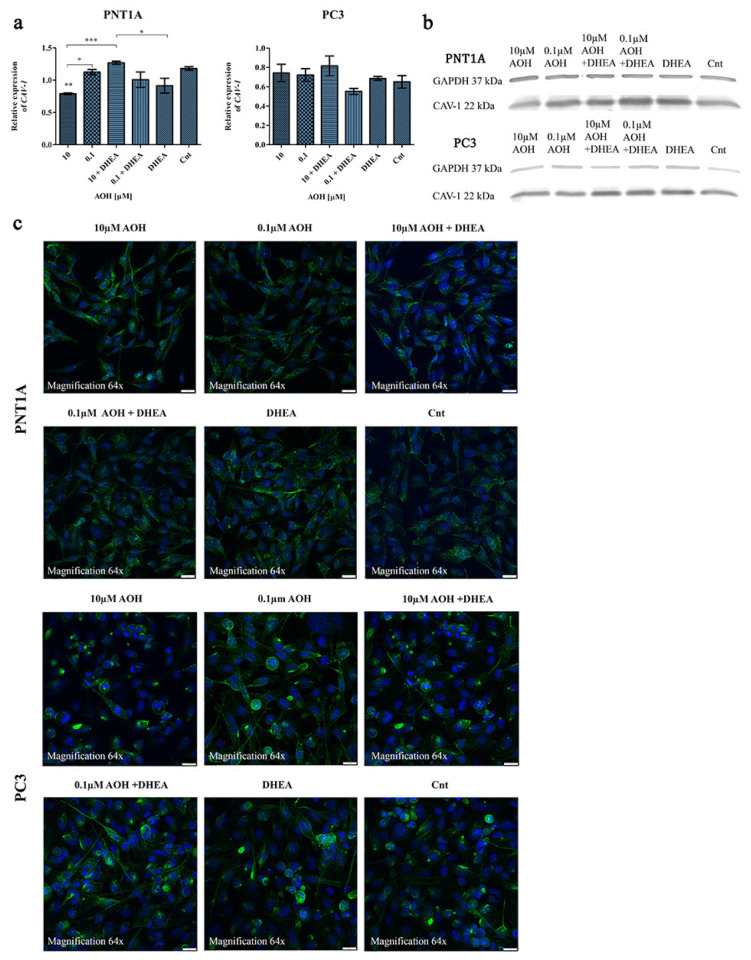
AOH affects the expression and localization of CAV-1 in prostate cells. Analysis of the relative expression of the *CAV-1* gene in normal and prostate cancer cell lines (**a**). One-way ANOVA was used for statistical analysis, and *p* < 0.05 was considered statistically significant. One-way ANOVA was used to perform statistical analysis of the results, and *p* < 0.05 was considered as statistically significant. * *p* < 0.05, ** *p* < 0.01, *** *p* < 0.001. The results are presented and mean ± SE. The results are expressed as the mean relative expression of three independent experiments. Representative results of Western blot analysis of CAV-1 expression (**b**). Observed different localizations of CAV-1 in cells from the confocal microscopy analysis performed in the sequence series of acquisition under the same microscope settings (**c**). AOH—alternariol, DHEA—dehydroepiandrosterone, Cnt—control, *CAV-1*—caveolin 1.

CAV-1 allows binding of the cholesterol and regulates the intracellular transport of cholesterol to and from the plasma membrane [26]. The structure of AOH resembles endogenous molecules such as cholesterol [10]; hence, this may provide the key for understanding its complex biological functions, including the modulation of the process of steroidogenesis. Therefore, during the evaluation of the effect of AOH on prostate cells’ steroidogenesis, we also decided to evaluate the possible changes in CAV-1 expression and localization, due to the known role of CAV-1 in prostate cancer [27]. Firstly, we evaluated the changes in the gene expression and observed that in PNT1A cells, 10 µM of AOH significantly reduced the expression of *CAV-1* as compared to the control (** *p* < 0.01) as well as 0.1 µM of AOH (* *p* < 0.05) and 10 µM of AOH + DHEA (*** *p* < 0.001) (Figure 4a). DHEA itself decreased the expression of *CAV-1,* and a similar effect was observed for 0.1 µM of AOH + DHEA as compared to 0.1 of µM AOH; however, the differences were not statistically significant. In case of PC3 cells, almost no changes in the expression of *CAV-1* were observed, besides the insignificant decrease upon 0.1 µM AOH + DHEA treatment. A similar tendency was observed for the protein expression of CAV-1 obtained in the Western blot analysis (Figure 4b and Table 1). For both cell lines, the expression of CAV-1 was slightly decreased after AOH treatment and increased after AOH + DHEA treatment. The altered expression might be associated with the different observed localization of AOH in cells (Figure 4c). In PNT1A cells, fluorescent staining of CAV-1 after AOH treatment showed a cell membrane and nuclear localization instead of predominantly cytosolic localization in control cells. In case of PC3 cells, a similar effect was observed for lower-dose AOH treatment rather than the higher dose of AOH.

### 2.4. AOH Modulates Cell Cycle and Apoptosis in Prostate Cells

**Figure 5:**
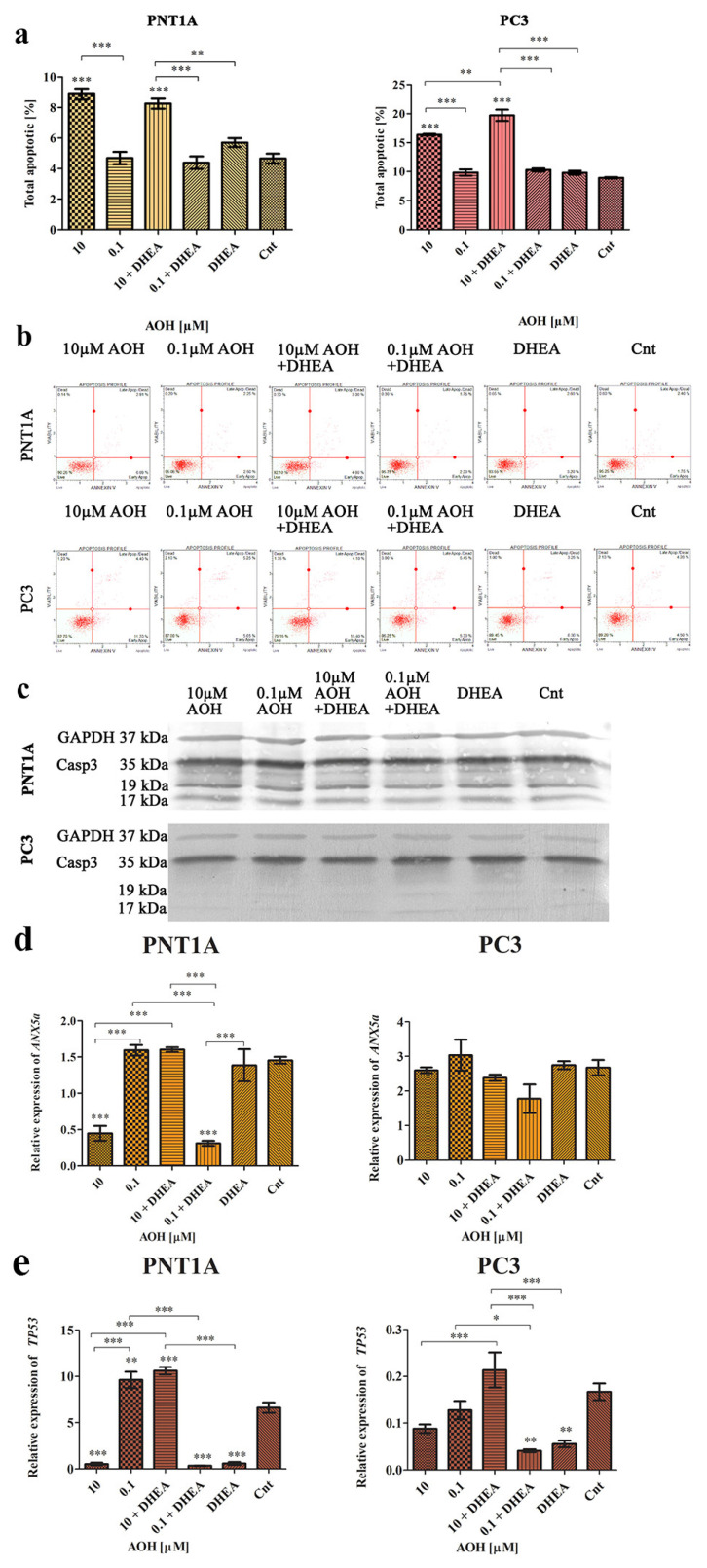
AOH, DHEA, as well as co-treatment induce apoptosis after 48 h of exposure in PC3 and PNT1A cells (**a**). Analysis of apoptotic cells based on flow cytometry with representative results (**b**). One-way ANOVA was used for statistical analysis. Results are presented and mean ± SE. * *p* < 0.05, ** *p* < 0.01, *** *p* < 0.001, as compared to the control. Representative results of Western blot analysis of *CASP3* (**c**). Analysis of the relative expression of *ANX5a* (**d**) and *TP53* genes in normal and prostate cancer cell lines (**e**). AOH—alternariol, DHEA—dehydroepiandrosterone, Cnt—control, Casp3—caspase 3.

Next, we evaluated the cell cycle and process of apoptosis in prostate cells, due to the fact that all of them are associated with the steroidogenesis process both in normal as well as cancer cells [28]. Firstly, the induction of apoptosis was evaluated with flow cytometry (Figure 5a,b), and it was revealed that in both normal as well as cancer cell lines, AOH at a dose of 10 µM induced a significant increase in the number of apoptotic cells (*** *p* < 0.001) as compared to the control. In the case of a lower tested dose (0.1 µM of AOH), no such effect was observed, similarly to DHEA treatment. Simultaneous treatment with AOH + DHEA resulted in a significant increase in the number of apoptotic PC3 cells as compared to AOH treatment alone (** *p* < 0.01) and DHEA (*** *p* < 0.001). In both cases, the increased number of apoptotic cells was associated with an increased number of early apoptotic cells rather than dead cells, as visible on flow cytometry histograms (Figure 5b). The increase in the number of apoptotic cells in PC3 cells corelated with the observed modulation of the expression of caspase 3 on the protein level (Figure 5c and Table 2), whereas in the case of PNT1A cells, the expression of caspase 3 was increased only in the 10 µM + DHEA treatment, as compared to non-treated cells. We also evaluated the expression of *ANXA5* (Figure 5d) and observed that in PNT1A cells, 10 µM of AOH caused a significant decrease in the expression of *ANXA5,* as compared to the control cells (*** *p* < 0.001), whereas 10 µM of AOH + DHEA resulted in no change in the expression as compared to the control. A contradictory effect was observed for a lower dose of AOH (Figure 5d). In the case of PC3 cells, modulation of *ANXA5* expression was not significant, although a similar trend was observed to a lower extent (Figure 5d). In the case of the expression of *TP53* in both cell lines, a similar effect was observed (Figure 5e). Treatment with 10 µM of AOH resulted in decreased expression, whereas the higher dose did not cause any change in expression as compared to the control in PC3 cells (** *p* < 0.01). In the case of *TP53,* we noticed that AOH led to an insignificant expression decrease in PNT1A cells, while the addition of DHEA resulted in a significant increase in expression in the case of a higher dose of AOH (*** *p* < 0.001) and a significant decrease in the expression in the case of a lower dose (* *p* < 0.05). DHEA itself caused a significant decrease in the expression of *TP53* as compared to control cells (** *p* < 0.01). A similar effect was observed in PC3 cells, but to a higher extent (Figure 5e). These changes might be explained by the fact that in the case of PC3 cells, more dead cells were observed as compared to PNT1A cells after AOH treatment, indicating that the induction of apoptosis by AOH is not a main effect of its action.

**Figure 6:**
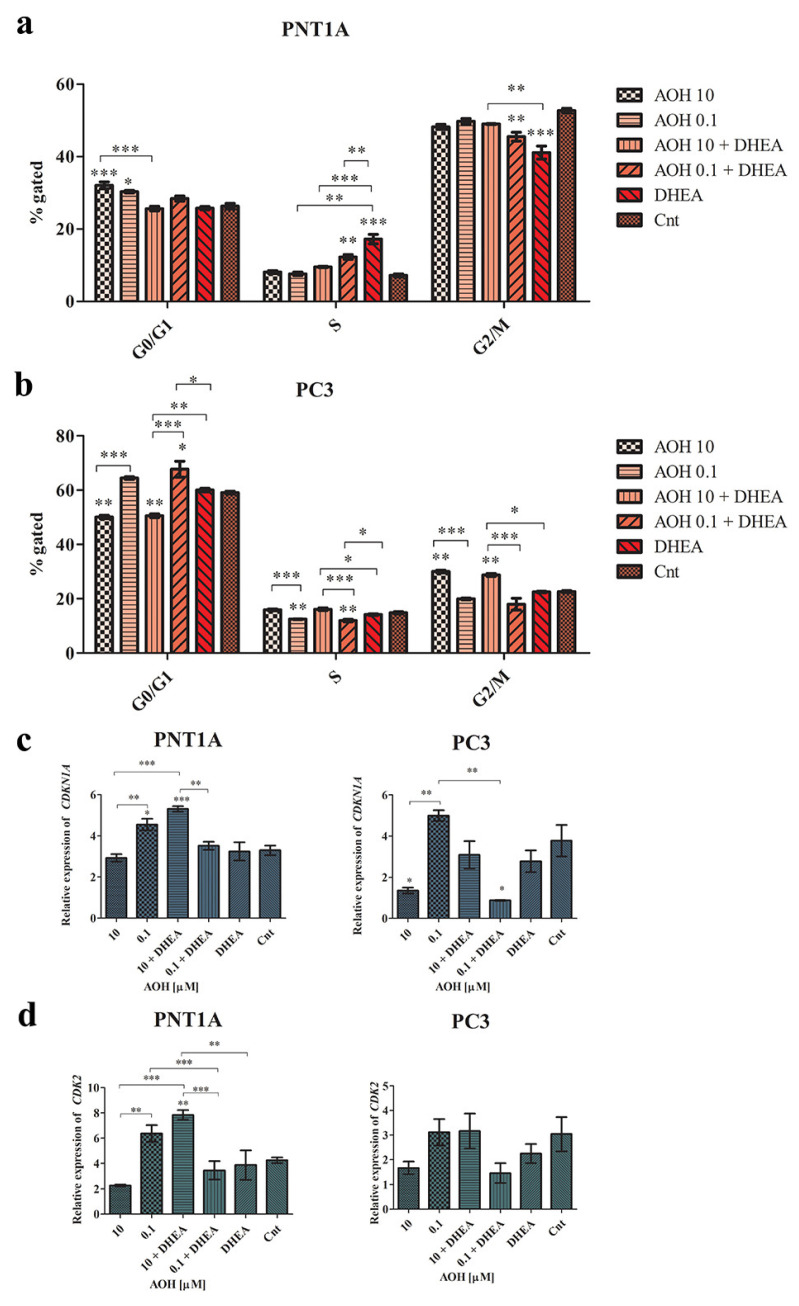
AOH, DHEA, as well as co-treatment induce changes in cell cycle progression after 48 h of exposure in PNT1A (**a**) and PC3 cells (**b**). Analysis based on the results obtained in flow cytometry. Analysis of the relative expression of *CDKN1A* (**c**) and *CDK2* genes in normal and prostate cancer cell lines (**d**). One-way ANOVA was used for statistical analysis. The results are presented as mean ± SE. * *p* < 0.05, ** *p* < 0.01, *** *p* < 0.001. AOH—alternariol, DHEA—dehydroepiandrosterone, Cnt—control. The results were acquired in three independent experiments.

We have also found that AOH differently modulated the cell cycle progression in normal and prostate cancer cells. In normal PNT1A cells, a significant increase in the number of G0/G1 cells was observed for both tested doses of AOH (*** *p* < 0.001 and * *p* < 0.05, respectively), compared to non-treated cells. DHEA as well as DHEA + AOH increased the number of cells in the S cell cycle phase, with a simultaneous decrease in the number of cells in the G2/M cell cycle phase (Figure 6a). The increase in the number of cells in the S phase of the cell cycle, as the well as the decrease in the G2/M cell cycle phase, was statistically significant for 0.1 µM of AOH + DHEA, as compared to 0.1 µM of AOH alone (** *p* < 0.01) and DHEA alone (** *p* < 0.01). In PC3 cells, a different effect was observed: AOH at a dose of 10 µM induced a significant increase in the number of cells in the G2/M cell cycle phase compared to the control (** *p* < 0.01). Simultaneous treatment with DHEA resulted in a similar effect. For a lower tested dose of AOH, a significant decrease in the number of cells in the S cell cycle phase was observed as compared to non-treated cells (** *p* < 0.01), with no significant increase in the number of G0/G1 cells (Figure 6b).

Next, the observed modulation of the cell cycle was compared with the expression of cell cycle regulators: *CDKN1A* and *CDK2.* A similar effect was observed in modulating the expression of *CDKN1A* as that of *TP53* expression, and a higher modulatory effect was observed in the PC3 cell line (Figure 6c). The observed changes in cell cycle progression might also be associated with modulation of the expression of *CDK2*, especially in the case of PNT1A cells, in which G0/G1 cell cycle arrest was observed. As suspected, 10 µM of AOH decreased, whereas 0.1 µM increased, *CDK2* expression, and the observed effect was significantly different (** *p* < 0.01). Similar to the other genes’ expression evaluated, the addition of DHEA to AOH treatment resulted in a significant contradictory effect, as observed for AOH alone (*** *p* < 0.001). In PC3 cells, a similar effect was observed, but not statistically significant (Figure 6d).

## 3. Discussion

The role of androgens in both physiological as well as pathological processes is already known, mainly because testosterone deprivation therapy is still the most effective strategy in PCa. Nevertheless, castration-resistant PCa still constitutes a major health problem and suggests that some patients re-express the AR [29]. One of the possible explanations might be the fact that the prostate itself might produce or support the steroidogenesis process. As previously observed, the presence of the transcript of all important enzymes for testosterone synthesis seems to confirm this hypothesis [30]. Moreover, we hypothesized that environmental pollutants or toxins which might influence the process of steroidogenesis can also affect the local steroidogenesis process in both normal as well as prostate cancer cells.

AOH is mainly considered as genotoxic due to its inhibitory effect on topoisomerase [31]. It was also reported as a weak estrogenic agent, mostly affecting Erβ [32] as well the AR agonist at high concentrations [33]. Although the observed effect of AOH on the steroidogenesis process is low, a more interesting fact is that AOH in the presence of other estrogenic agents is able to trigger a myriad of different effects [34]. Therefore, in this study, we firstly evaluated the effect of AOH itself on local prostate steroidogenesis, but also in combination with a known androgenic agent (DHEA). The concentrations chosen for the study are subtoxic to the cells, based on our previous and current research studies [21]. Based on the EFSA report, the contamination of AOH in food products ranges between 2.5 μg/kg in tomatoes and 39.7 μg/kg in oats [35]. Taking into consideration the recovery ratio of *Alternaria* toxin mixtures [36], the exposition of cells to the high nM to low µM concentration range is possible, in line with the experimental design of this study. We have found that AOH alone did not affect the production of testosterone, estradiol, or progesterone, but the combination with DHEA exacerbated its effect in normal as well as prostate cancer cells. 

The abovementioned effect was associated with modulated expression of most important steroidogenic enzymes: *CYP11A1*, *STAR*, *CYP17A1*, *HSD3B2*, as well as *AR* and *ESR2.* Our observation is in line with the previous reports suggesting the endocrine modulatory effect of AOH in cells. Kalayou et al. observed that AOH increased the expression of *HSD3B* and *CYP21A2* in the human adenocarcinoma cell line H295R [9]. The same cell line was used by Frizzell et al. to assess the endocrine-disrupting effect of AOH, and they found a modulation of the expression of *CYP11A1* and *CYP17* [37]. In our study, AOH itself increased the expression of the *StAR* transcript responsible for cholesterol transport to the inner mitochondrial membrane, *HSD3B2*, as well *AR* and *ESR2*, whereas a contradictory effect was observed for the *CYP11A1* transcript, responsible for conversion of cholesterol to pregnolone by mitochondrial enzymes, and *CYP17A1,* that participates in testosterone production. One of the possible explanations for the modulation of steroidogenesis is the fact that AOH has been postulated to possess a similarity to cholesterol and possibly intercalate the cholesterol-rich membrane domains, e.g., caveolae [10]. To elaborate on that, we evaluated the expression of *CAV-1* and its cellular localization after treatment with AOH. Our results were different from those observed by Del Favero et al. in THP-1 cells, but a modulation of the expression and localization was found after AOH treatment [10]. On the other hand, a higher tested dose of AOH in another study (10 µM) resulted in a decrease in the expression of CAV-1 in intestinal cells, which is in line with our results [38]. 

In previous studies, AOH was reported to modulate the process of apoptosis and the cell cycle process in cells, i.e., human colon carcinoma, murine hepatoma, human colorectal adenocarcinoma, and Abelson murine leukemia [39,40]. Kalayou et al. observed that AOH influences the proliferation and cell cycle progression of cells depending on the dose, with arrest in the G0/G1 or G2/M cell cycle phases [9]. In this study, we observed that AOH modulated the cell cycle progression differently in the different prostate cell lines. The findings of Kalayou might explain the different cellular effects of PNT1A and PC3 cells, also observed in this study. The induction of cell cycle arrest was associated with modulation of the genes responsible for cell cycle progression, *CDKN1A*, *CDK2,* and *TP53*. Solhaug at al. previously suggested that modulation of the cell cycle by AOH is associated with phosphorylation of histone H2AX, activation of p53, and increased expression of p21, cyclin B1, and other cell cycle regulators, although they tested higher doses of AOH than in this study (60 µM) [3].

Nevertheless, in both cell lines, the higher tested dose induced apoptosis in cells, and DHEA did not abolish that effect. Modulation of cell cycle progression by AOH and apoptosis was also observed in Caco-2 cells, in which AOH induced cell cycle arrest in the G2/M cell cycle phase with simultaneous induction of apoptosis and necrosis [40]. The concentration range in that study was higher than in our study, indicating the switch between apoptosis and necrosis in cells. In the HCT116 cell line, AOH also induced necrosis/apoptosis with modulation of the expression of caspase 3 [41], similar to our observations, but the suggested mechanism was associated with DNA damage, and therefore with AOH genotoxicity. Here, we report that the induction of apoptosis and cell cycle progression could be different in normal and cancer cell lines, even for the same doses of the tested mycotoxins, and might be associated with modulation of steroidogenesis, not only genotoxicity.

To the best of our knowledge, this is the first study to evaluate the effect of AOH as well as the combinatory effect of AOH and DHEA on a local steroidogenesis process in both normal and prostate cancer cells. We observed that the effect of AOH on the process of steroidogenesis was low, but in combination with other steroidogenic agents, it triggered significant changes in both normal and cancer cells. Moreover, this study showed that AOH might also affect the apoptosis cell cycle in cells, which consequently might exacerbate its effect on steroidogenesis in cells.

## 4. Materials and Methods

### 4.1. Cell Culture and Experimental Treatments

The normal human prostate epithelial cell line PNT1A and the prostate adenocarcinoma cell line PC3 were obtained from the European Collection of Authenticated Cell Cultures (ECACC) (Sigma-Aldrich, Saint Louis, MO, USA) and maintained in a humidified incubator (37 °C, 5% CO_2_). The cells were cultured in RMPI medium with 10% of heat-inactivated fetal bovine serum (FBS), 2 mM of L-glutamine, 1 mM of sodium pyruvate, 10 mM of HEPES, and 1% of PenStrep (5000 units/mL of penicillin and 5000 µg/mL of streptomycin). All media and supplements were purchased from Thermo Fisher Scientific Inc., Waltham, MA, USA. 

Alternariol (AOH) was purchased from Sigma-Aldrich, Saint Louis, USA, and dissolved in dimethyl sulfoxide (DMSO) to obtain a stock solution. The dehydroepiandrosterone (DHEA) stock solution (Avanti Polar Lipids, Inc., Alabaster, AL, USA) was prepared in ethanol. Stocks were dissolved in experimental medium each time before use. In the experimental model, cells were treated with 0.1 or 10 µM of AOH and/or 100 nM of DHEA for 48 h. Non-treated cells were used as a control. The experimental doses of AOH and DHEA were based on our previous observations and research [19,20]. All experiments were performed in triplicates.

### 4.2. Cell Viability Assay

The cell viability was determined by the MTT (3-(4,5-dimethylthiazol-2-yl)-2,5-diphenyltetrazolium bromide) (Merck Millipore, Burlington, MA, USA) assay according to the manufacturer’s instructions. The cells were seeded in a 96-well plate at the concentration of 1 × 10^5^ in 100 µL of culture media. The following day, the culture medium was changed to the experimental medium in the following combinations: 0.1 µM of AOH, 10 µM of AOH, 0.1 µM of AOH + 100 nM of DHEA, and 10 µM of AOH + 100 nM of DHEA for 48 h. Four hours before the end of the incubation time, 5 mg/mL of MTT solution was added to the each well (10 µL per well). The formed formazan crystals were dissolved in DMSO (100 µL of solvent added per well). The absorbance at 570 nm was measured using an ELX 80IU microplate reader (BioTek, Winooski, VT, USA).

### 4.3. Annexin V Staining Assay 

The flow cytometry assays, Muse^®^ Annexin V and Dead Cell Kit (Merck Millipore, Burlington, MA, USA), were conducted to assess the number of apoptotic cells. The cells were seeded on a 6-well plate at density of 3 × 10^5^/well. After reaching ca. 90% of confluence, the experimental treatment was performed. Cells were exposed to 0.1 µM of AOH, 10 µM of AOH, 0.1 µM of AOH + 100 nM of DHEA, and 10 µM of AOH + 100 nM of DHEA. After 48 h, the cells were detached and suspended in 100 µL of culture medium. Assays were performed according to the manufacturer’s instructions. The cells were analyzed on the Muse™ Cell Analyzer (Merck Millipore, Burlington, MA, USA). Experiments were carried out in triplicates.

### 4.4. Cell Cycle

The Muse^®^ Cell Cycle Assay Kit (Merck Millipore, Burlington, MA, USA), based on propidium iodide (PI) staining, was used to evaluate the percentage of cells in the G0/G1, S, and G2 phases of the cell cycle. The cells were seeded on a 6-well plate at a density of 3 × 10^5^/well. After reaching ca. 90% of confluence, the experimental treatment was performed. Cells were exposed to 0.1 µM of AOH, 10 µM of AOH, 0.1 µM of AOH + 100 nM of DHEA, and 10 µM of AOH + 100 nM of DHEA. After 48 h, cells were detached and suspended in 100 µL of culture medium. The assays were performed according to the manufacturer’s instructions. The cells were analyzed on the Muse™ Cell Analyzer (Merck Millipore, Burlington, MA, USA). The experiments were carried out in triplicate.

### 4.5. Steroid Assays

The enzyme-linked immunosorbent assay (ELISA) was used to assess the concentration of steroid hormones: progesterone (sensitivity 8.57 pg/mL), testosterone (sensitivity 5.67 pg/mL), and 17-β-estradiol (sensitivity 28.5 pg/mL). Cells were seeded on a 6-well plate (3 × 10^5^/well) to reach a satisfactory confluence of ca. 90%. After 24 h, the culture medium was changed to experimental media containing 1 µM of AOH, 10 µM of AOH, 1 µM of AOH + 100 nM of DHEA, and 10 µM of AOH + 100 nM of DHEA for 48 h. The 100 nM DHEA treatment was used as a positive control. The media collected from the experiments were used to perform ELISA assays (Enzo Chemicals Inc., Farmingdale, NY, USA) according to the manufacturer’s instructions. The absorbance was measured at 405 nm. All the assays were run in duplicates.

### 4.6. RNA Extraction and Real-Time Quantitative Polymerase Chain Reaction (RT-qPCR)

Total RNA was extracted with the use of TRIzol reagent (Thermo Fisher Scientific Inc., Waltham, MA, USA). Cells were cultured in 60 nm Petri dishes at a density of 2 × 10^5^ cells per dish. After reaching the confluence of 80%, the experimental treatment was performed. The culture medium was replaced with the experimental ones in the following combinations: 1 µM of AOH, 10 µM of AOH, 1 µM of AOH + 100 nM of DHEA, and 10 µM of AOH + 100 nM of DHEA, while 100 nM of DHEA was used as a positive control. After 48 h, the medium was removed, and the RNA isolation procedure was performed according to the manufacturer’s instructions. The BioDrop DUO spectrophotometer (BioDrop, Cambridge, UK) was used to measure the RNA concentration. cDNA synthesis was performed using Prom RT-IITM reverse transcriptase (Promega, Madison, WI, USA). Analyzed target genes included: cytochrome P450 family 11 subfamily A member 1 (*CYP11A1),* cytochrome P450 family 17 subfamily A member 1 (*CYP17A1*)*,* 3 beta- and steroid delta-isomerase 2 (*HSD3B2*)*,* hydroxysteroid 17-beta dehydrogenase 2 (*HSD17B2*), steroidogenic acute regulatory protein (*StAR*), annexin 5 (*ANXA5*), estrogen receptor 2 (*ESR2*), androgen receptor (*AR*), caveolin 1 (*CAV-1*), cyclin-dependent kinase inhibitor 1 *(CDK1N1A*)*,* cyclin-dependent kinase 2 (*CDK2*), and tumor protein p53 (*TP53*). As the calibrator, we used the human reference RNA (Stratagene, San Diego, CA, USA). Primers’ design and validation were performed with Primer BLAST software. Sequences and product sizes are presented in Table 3. Housekeeping genes were used for relative expression normalization as calibrators: ribosomal protein S17 (*RPS17*), ribosomal protein P0 (*RPLP0*), and histone H3.3A (*H3F3A*). The obtained values were calculated using the ΔΔCt method. Melting curve analyses were performed to verify the identity of the product for each reaction.

### 4.7. Western Blot

Cells were cultured on Petri dishes (100 nm) at a density of 3 × 10^5^ to reach a satisfactory confluence of 90%, and then the experimental treatments were performed. For the isolation of total protein extracts, RIPA protein extraction buffer was used, supplemented with protease and phosphatase inhibitor cocktails (Sigma-Aldrich) and 1 mM of PMSF (Sigma Aldrich, Saint Louis, MO, USA). Protein concentration was measured using Direct Detect^®^ (Merck Millipore, Burlington, MA, USA).

For gel electrophoresis, 10 µg of protein was mixed with the Laemmli Lysis buffer and heated for 5 min at 100 °C. Proteins were separated with 12.5% SDS-polyacrylamide gels and transferred to PVDF membranes (400 mA, 110 min) (Merck Millipore, Burlington, MA, USA) with wet transfer. The protein visualization after electrophoresis was performed with the use of 0.1% Panceau-S (Sigma Aldrich, Saint Louis, MO, USA) in 1% acetic acid. Then, 5% nonfat milk in TBST, for 1 h at RT, was used to block membranes, which were then incubated overnight at 4 °C with selected primary antibodies according to the manufacturer’s instructions: CAV-1 (sc-894, Santa Cruz Biotechnology, Dallas, TX, USA), GAPDH (sc-59540, Santa Cruz Biotechnology, Dallas, TX, USA), and Cleaved Caspase-3 (mAb #9664, CST, Danvers, MA, USA). The following day, membranes were washed with TBST buffer (3 × 5 min) and incubated with the solution of secondary antibody (1:15,000, in 1% nonfat milk in TBST) conjugated with alkaline phosphatase (A3812, Sigma Aldrich, Saint Louis, MO, USA). After incubation, membranes were washed again with TBST buffer (3 × 5 min). Protein bands were visualized using Novex^®^ AP Chromogenic Substrate (Life Technologies, Carlsbad, CA, USA).

### 4.8. Immunohistochemistry Staining and Confocal Microscopy

The cells were seeded on 8-well chamber slides (NuncTM Lab-TekTM II Chamber SlideTM System/Thermo Fisher Scientific Inc, Waltham, MA, USA). Once the slides reached 80% confluence, experimental treatments were performed for 48 h. Cell fixation was performed in 70% ice-cold methanol for 15 min in a freezer. The cells were washed three times in DPBS. Subsequently, cells were immersed in the blocking buffer (5% FBS, 0.3% Triton X-100 in DPBS 1X) for 1 h. Incubation with the primary antibody CAV-1 (sc-894, Santa Cruz Biotechnology, Dallas, TX, USA) was performed overnight (1:100, 1% BSA, 0.3% Triton X-100 in DPBS 1X). The following day, cells were washed three times with DPBS and incubated for 1.5 h with a secondary antibody (1:400, Alexa Fluor Plus^®^ 488, goat anti-rabbit, #A32731, Thermo Fisher Scientific Inc, Waltham, MA, USA). Upon completed incubation, the final washing step was performed (three times in DPBS 1X), and the mounting medium, Fluoroshield with DAPI (F6057, Sigma, St. Louis, MO, USA), was used to preserve the fluorescence of cell specimens. Confocal fluorescence imaging was performed with the use of Olympus iXplore SpinSR ScanR (Olympus, Tokyo, Japan).

### 4.9. Statistical Analysis

Statistical data analysis was performed with the GraphPad Software (GraphPad Software, San Diego, CA, USA), using one-way ANOVA. The results are expressed as mean ± SE, and *p* < 0.05 was considered statistically significant.

## Figures and Tables

**Table 1:** Effects of AOH, DHEA, and co-treatment of AOH and DHEA on the protein expression of CAV-1. The results are presented as fold changes in the expression. AOH—alternariol, DHEA—dehydroepiandrosterone, Cnt—control, CAV-1—caveolin 1.

Cell Line	Treatment	CAV-1
PNT1A	10 μM AOH	0.62
	0.1 μM AOH	1.06
	100 nM DHEA	1.08
	10 μM AOH + 100 nM DHEA	1.61
	0.1 μM AOH + 100 nM DHEA	1.48
	Cnt	1.00
PC3	10 μM AOH	0.67
	0.1 μM AOH	0.44
	100 nM DHEA	0.95
	10 μM AOH + 100 nM DHEA	0.62
	0.1 μM AOH + 100 nM DHEA	0.57
	Cnt	1.00

**Table 2:** Effects of AOH, DHEA, and co-treatment of AOH and DHEA on the protein expression of Casp3 and Cleaved Casp3. The results are presented as fold changes in the expression. AOH—alternariol, DHEA—dehydroepiandrosterone, Cnt—control, Casp3—caspase 3.

Cell Line	Treatment	Casp3	Cleaved Casp3
PNT1A	10 μM AOH	0.94	0.70
	0.1 μM AOH	0.89	0.66
	100 nM DHEA	1.33	1.03
	10 μM AOH + 100 nM DHEA	0.92	0.71
	0.1 μM AOH + 100 nM DHEA	1.31	0.80
	Cnt	1.00	1.00
PC3	10 μM AOH	2.15	1.83
	0.1 μM AOH	1.07	1.42
	100 nM DHEA	1.51	1.92
	10 μM AOH + 100 nM DHEA	0.98	0.98
	0.1 μM AOH + 100 nM DHEA	0.99	1.38
	Cnt	1.00	1.00

**Table 3:** Primers used in RT-qPCR.

Gene	Sequence (5′-3′)	Product Size (bp)
*CYP11A1*	For CCAGAACGATTCCTCATCC	126
	Rev CATCACCTCCTGGTTCAG	
*CYP17A1*	For GAAGTTATCATCAATCTGTGGG	119
	Rev ACTGACGGTGAGATGAGC	
	Rev AAGATGTCTGGTTTGATGAGGAG	
*HSD3B2*	For CTTGGTGTCACTCACAGAGAG	128
	Rev GTAGATGAAGACTGGCACACTG	
	Rev CACCTCCAATTGTGACATAA	
*STAR*	For CATGGAGAGGCTCTATGAAGA	128
	Rev CAGCCAGCTCGTGAGTAAT	
*ANXA5*	For ACCCTCTCGGCTTTATGATGCT	116
	Rev TGGCTCTCAGTTCTTCAGGTGT	
	Rev TGCTGGACAGAAATGTGTACACTCCAGA	
*ESR2*	For ACACCTGGGCACCTTTCTCCTTTA	90
	Rev TCTTGCTTCACACCAGGGACTCTT	
*AR*	For GGGAGGTTACACCAAAGGGC	102
	Rev AGAGACAGGGTAGACGGCAG	
*CAV-1*	Rev GAACTTGAAATTGGCACCAGG	139
	For ACCCACTCTTTGAAGCTGTTG	
*CDK1N1A*	Rev CTGAGACTAAGGCAGAAGATGT	133
	For GACAGATTTCTACCACTCCAA	
*CDK2*	Rev GAGCAGAGGCATCCATGAATT	126
	For TGCTTAAGGAGCTTAACCATCC	
*TP53*	Rev TTTATGGCGGGAGGTAGA	102
	For TTGGAACTCAAGGATGCC	
	For GCAAGACTGTTAGCCCTCAA	
*RPLP0*	For ACGGATTACACCTTCCCACTTGCTAAAAGGTC	69
	Rev AGCCACAAAGGCAGATGGATCAGCCAAG	
*RPS17*	For AAGCGCGTGTGCGAGGAGATCG	87
	Rev TCGCTTCATCAGAT GCGTGACATAACCTG	
*H3F3A*	For AGGACTTTAAAAGATCTGCGCTTCCAGAG	74
	Rev ACCAGATAGGCCTCACTTGCCTCCTGC	

*RPS17*—ribosomal protein S17; *RPLP0*—ribosomal protein P0; *H3F3A*—histone H3.3A; *CYP11A1*—cytochrome P450 family 11 subfamily A member 1; *CYP17A1*—cytochrome P450 family 17 subfamily A member 1; *HSD3B2*—3 beta- and steroid delta-isomerase 2; *STAR*—steroidogenic acute regulatory protein; *ANXA5*—annexin 5; *ESR2*—estrogen receptor 2; AR—androgen receptor; *CAV-1*—caveolin; *CDK1N1A*—cyclin-dependent kinase inhibitor 1; *CDK2*—cyclin-dependent kinase 2; *TP-53*—tumor protein p53.

## Data Availability

The raw data or unpublished data that support the findings of this study are available upon request from the corresponding author.
